# Risk prediction of pediatric intensive care unit admission in children with respiratory syncytial virus infection using interpretable machine learning

**DOI:** 10.3389/fmed.2026.1856930

**Published:** 2026-07-08

**Authors:** Junyu Dong, Jingwen Ni, Mengxin Zhao, Zhihui Du, Kenan Fang

**Affiliations:** PICU of Luoyang Maternal and Child Health Hospital, Luoyang, Henan, China

**Keywords:** children, machine learning, pediatric intensive care unit, respiratory syncytial virus, risk prediction

## Abstract

**Background:**

Respiratory syncytial virus (RSV) is a major cause of pediatric acute lower respiratory infection. Early prediction of pediatric intensive care unit (PICU) transfer may support clinical decision-making and resource allocation. We aimed to develop and temporally validate an interpretable machine learning model for predicting PICU admission in hospitalized children with RSV.

**Methods:**

We included children aged 29 days–18 years with laboratory-confirmed RSV at a single center (January 2023–April 2025). The prediction target was PICU admission within 48 h of hospital admission. Internal validation was performed using random split-sample (80% training / 20% internal test set) and 10-fold cross-validation during hyperparameter tuning. The development cohort (January 2023–March 2025; *n* = 1,606, 209 PICU admissions) was randomly split into training (80%, *n* = 1,285, 167 events) and internal test (20%, *n* = 321, 42 events) sets. A separate temporal external validation cohort (April 2025; *n* = 94, 12 events) from the same institution was used for temporal external validation. Ten machine learning algorithms were trained using day-one clinical and laboratory variables and evaluated by AUROC, average precision, classification metrics, calibration curves, and decision curve analysis. SHAP was used for interpretation.

**Results:**

Among 1,606 children, 209 required PICU admission. Final predictors included dyspnea, serum ferritin, wheezing, immunoglobulin G, interleukin-6, preterm birth, and personal history of wheezing. Random forest performed best among ten algorithms, with AUROC 0.94, average precision 0.87, accuracy 0.95, precision 0.86, recall 0.76, and F1 score 0.81 in the internal test set. In the temporal external validation cohort, the model achieved an AUROC of 0.92, average precision of 0.82, accuracy of 0.95, precision of 0.94, recall of 0.68, and F1 score of 0.79. Calibration plots, decision curve analysis, and SHAP analysis suggested acceptable calibration, potential clinical utility, and interpretability.

**Conclusion:**

We developed an interpretable machine learning model to predict PICU transfer in children with RSV. The random forest model performed well, but development estimates may remain optimistic without bootstrap-based optimism correction, and the small temporal external validation cohort limits precision. Further geographical, institutional, and healthcare-system validation is needed before clinical implementation.

## Introduction

Respiratory syncytial virus is a leading cause of acute lower respiratory infections in children aged 0–5 years (1). Annually, approximately 33 million cases of RSV-related lower respiratory infections are reported globally, with 3.6 million requiring hospitalization, and around 5% of these hospitalized children being admitted to pediatric intensive care units (PICU) (2). Following the COVID-19 pandemic, non-seasonal RSV outbreaks have increased significantly (3–5), resulting in higher rates of severe cases, PICU admissions, and longer hospitalization durations, further exacerbating the strain on pediatric intensive care resources (3, 6–8). Therefore, early identification of high-risk children who may require PICU care is crucial for timely intervention and optimal resource management.

A previous study (9) have predominantly utilized perinatal or early postnatal factors—such as delivery conditions and congenital abnormalities—to predict the likelihood of severe illness and ICU admission should infants become infected with RSV in the future, which have primarily served population-level risk screening and the planning of preventive interventions, including monoclonal antibody administration and vaccine prioritization, particularly for preterm infants or those with underlying health conditions. However, there is a lack of research focused on children already infected with RSV, aiming to predict the need for PICU admission based on clinical data available at the time of presentation. Existing model (9) are insufficient for guiding early clinical decision-making during emergency visits or initial hospitalization. This limitation is especially evident during peak RSV seasons, when pediatric critical care resources are limited and PICU beds are frequently at capacity. Therefore, there is an urgent need to develop accurate, real-time risk prediction tools to support clinical judgment and optimize the allocation of intensive care resources.

Machine learning techniques offer significant advantages in addressing these challenges, as they can capture non-linear relationships and high-dimensional interactions in data, providing improved prediction accuracy. Despite their predictive advantages, the “black-box” nature of many machine learning models makes it difficult for clinicians to understand the rationale behind their predictions, which may limit clinical utility (10). SHAP provides a transparent feature contribution analysis, helping clinicians understand the influence of each variable on the model’s predictions, thereby enhancing the interpretability and trustworthiness of the model (11).

In hospitalized children with RSV infection or bronchiolitis, clinical deterioration requiring escalation to pediatric intensive care may occur after initial evaluation in the emergency department or general pediatric ward. Previous studies have described ICU/PICU transfer or critical care escalation among children hospitalized with RSV infection or bronchiolitis, and clinical guidance emphasizes monitoring respiratory rate, work of breathing, oxygen saturation, and auscultatory findings during hospitalization (12, 13). Therefore, a clinically useful prediction model should rely on information available at or shortly after hospital admission and help identify children at increased risk before the need for intensive care becomes unequivocally apparent. The present model was designed to support early risk stratification within this clinical window, rather than to replace immediate clinical judgment in children who already clearly meet PICU admission criteria at presentation. The model was designed to support early risk stratification in children who are initially admitted to the general pediatric ward and for whom the need for PICU transfer is not immediately obvious at presentation.

Therefore, this study utilized data from children hospitalized with laboratory-confirmed RSV infection at Luoyang Maternal and Child Health Hospital to develop and validate an interpretable machine learning model using both internal validation (random split-sample and cross-validation) and temporal external validation for predicting actual PICU transfer within 48 h of hospital admission.

## Materials and methods

### Study design

This retrospective, single-center cohort study consecutively included children aged 29 days–18 years who were hospitalized with laboratory-confirmed RSV infection at Luoyang Maternal and Child Health Hospital between 1 January 2023 and 30 April 2025. The primary outcome was actual transfer to the pediatric intensive care unit (PICU) within 48 h of hospital admission, as recorded in the electronic medical records. The intended use of the model was early risk stratification after hospital admission. The model is intended for children hospitalized with RSV infection who do not clearly meet PICU admission criteria at the time of hospital admission (i.e., those initially managed in the general pediatric ward or under observation). The goal is to identify patients at risk of subsequent deterioration within 48 h, before the need for intensive care becomes unequivocally apparent. Candidate predictors were restricted to variables available on the first day of hospitalization and before the decision regarding PICU transfer, so that the model reflected information available during the early clinical decision-making window.

Patients admitted between 1 January 2023 and 31 March 2025, constituted the development cohort (*n* = 1,606). This cohort was randomly divided into a training set (80%, *n* = 1,285) and an internal test set (20%, *n* = 321), representing random split-based internal validation. In addition, 10-fold cross-validation was performed during hyperparameter tuning (on the training set only). Patients admitted between 1 April and 30 April 2025, constituted a temporal external validation cohort (*n* = 94). This cohort was derived from the same hospital but from a non-overlapping, subsequent time period and was not used in any model development step; therefore, it represents temporal external validation. For clarity, we used the following validation terminology in this study. “Statistical validation” or “model performance evaluation” refers to the assessment of model performance using discrimination, calibration, threshold-dependent classification metrics, and decision curve analysis. “Internal validation” (as performed in this study) refers to model evaluation using resampling or data splitting within the development data, such as split-sample validation or cross-validation. “Temporal external validation” refers to model evaluation using patients from a later, non-overlapping time period at the same institution. According to standard prediction model methodology (TRIPOD, TRIPOD-AI) (14, 15), external validation encompasses evaluation in independent new patients with meaningful separation from the development data that may be temporal, geographical, institutional, population-based, or healthcare-system-based. “Geographical external validation” (not performed) refers to evaluation using patients from a different institution, geographical setting, healthcare system, population, or clearly independent clinical environment. “Validation using another dataset” is a descriptive phrase, and its classification depends on the source, independence, time period, and clinical context of that dataset. The study protocol is available from the corresponding author upon reasonable request. This study was not registered in a clinical trial registry because of its retrospective, prediction-model nature.

All data preprocessing procedures, including feature selection and missing data imputation, were performed using the training set only to minimize data leakage. Missing data were imputed using the k-nearest neighbors (KNN) algorithm fitted on the training set, and the same imputation parameters were then applied to the internal test set and the temporal external validation cohort. Clinical and laboratory data were extracted from the hospital’s electronic medical records and anonymized before analysis. The study protocol was approved by the Ethics Committee of Luoyang Maternal and Child Health Hospital (Approval No: KY2022021401.0), which waived the requirement for informed consent owing to the retrospective design and data anonymization.

### Patient selection

Eligible patients were children hospitalized at Luoyang Maternal and Child Health Hospital between 1 January 2023 and 30 April 30, 2025, who met the following criteria.

Inclusion criteria:

(1) Age between 29 days and 18 years;

(2) The diagnosis of RSV infection was confirmed by respiratory specimen nucleic acid or rapid antigen testing (16), The choice of testing method was mainly determined by test availability and routine clinical workflow for children with clinically suspected RSV infection. Both methods were used during the study period as part of routine clinical care;

(3) Hospitalized for treatment with complete electronic medical record data.

Exclusion criteria:

(1) Presence of severe underlying conditions that would necessitate intensive care irrespective of the RSV infection. These included acute, life-threatening, or end-stage non-respiratory conditions such as major trauma (including fractures, extensive burns, etc.), active malignancies requiring intensive chemotherapy or causing organ failure, or end-stage renal disease. These conditions were excluded because the primary indication for PICU admission would likely be the underlying condition itself, potentially confounding the prediction model for RSV-specific severity;

(2) Missing key information (e.g., clinical manifestations, laboratory results, or primary outcomes missing in more than 10% of cases), ensuring data integrity;

(3) Lack of core laboratory indicators (such as blood gas analysis, white blood cell count, etc.), which would prevent participation in model training and validation.

### PICU admission criteria

Pediatric intensive care unit admission was defined as actual transfer to the PICU within 48 h of hospital admission, as recorded in the electronic medical records. Outcome status was determined from documented PICU transfer records during data extraction by the study investigators. Owing to the retrospective design and the use of routinely collected clinical records, blinded outcome assessment was not feasible. In our institution, PICU transfer was generally based on the following clinical criteria:

(1). Recurrent apnea or significant respiratory fatigue requiring stimulation or assisted ventilation for recovery (17);

(2). Refractory hypoxemia, defined as SpO_2_ remaining below 90% despite high-flow nasal cannula therapy (with flow rates ≥ 2 L/kg/min and FiO_2_ ≥ 0.40 for <18 years) in the general ward (18);

(3). Severe or worsening respiratory distress, characterized by a persistent respiratory rate > 70 breaths/min, pronounced chest retractions, grunting, or cyanosis, with no improvement after ≥6 h of standard supportive care;

(4). Hypercapnia and/or acidosis, indicated by PaCO_2_ > 55 mmHg or pH < 7.30 (19);

(5). Requirement for advanced respiratory support beyond ward capabilities, including HFNC failure or anticipated need for CPAP/BiPAP or endotracheal intubation;

(6). Hemodynamic or neurologic instability, such as shock requiring vasopressors, altered consciousness, or seizures;

(7). Presence of high-risk comorbidities known to increase susceptibility to severe RSV infection in combination with any of the above features (Criteria 1–6). These chronic conditions included prematurity (<32 weeks gestation), corrected age < 48 weeks, significant congenital heart disease, chronic lung disease, neuromuscular disorders, or immunodeficiency (20). Patients with these comorbidities were included in the study, and their PICU admission was determined by the severity of the acute RSV illness manifesting as physiological instability (meeting Criteria 1–6) (20);

Patients who meet all inclusion criteria and none of the exclusion criteria are labeled according to the above PICU admission standards, forming the basis for subsequent model development and validation.

### Data collection

All predictive variables were extracted from the electronic medical records on the first day of hospitalization and were categorized into four groups. Demographic characteristics included age in months and sex. Medical history included preterm birth (<37 weeks of gestation), personal history of atopy (yes/no), personal history of wheezing (yes/no), and breastfeeding (yes/no). Personal history of wheezing was defined as a documented history of previous wheezing episodes before the current hospitalization, based on caregiver report and information recorded in the electronic medical record by the attending pediatrician. Because more detailed information, such as the number of previous wheezing episodes, prior hospitalization for wheezing, or physician-diagnosed recurrent wheezing illness, was not consistently available in the retrospective records, this variable was coded as a binary variable (yes/no). Clinical characteristics at admission included fever (yes/no), cough (yes/no), wheezing (yes/no), and dyspnea (yes/no). These clinical variables were documented by attending pediatricians using a standardized clinical assessment form and were extracted directly from structured fields in the electronic health record system to ensure consistency and support automated, real-time prediction. All candidate predictors were recorded as part of the patient’s initial assessment and were available before the decision regarding PICU transfer. Therefore, the model was developed using information available at the time of early clinical decision-making. Laboratory indices were obtained from the first blood specimen after admission and included complete blood count parameters (white blood cell count, absolute neutrophil count, absolute lymphocyte count, hemoglobin, and platelet count), inflammatory and iron metabolism markers (C-reactive protein, procalcitonin, serum ferritin, and interleukin-6), liver and muscle enzymes (alanine aminotransferase, aspartate aminotransferase, creatine kinase, creatine kinase myocardial band, and lactate dehydrogenase), renal function and electrolytes (urea, creatinine, uric acid, sodium, and calcium), coagulation parameters (prothrombin time, activated partial thromboplastin time, D-dimer, and antithrombin III), and immunoglobulins (immunoglobulin A, immunoglobulin M, and immunoglobulin G). Interleukin-6, serum ferritin, and immunoglobulin G were measured from the first blood specimen after admission according to routine inpatient clinical practice in our institution. These measurements were available before the decision regarding PICU transfer.

### Model construction and evaluation

The study employed ten mainstream machine learning algorithms to develop predictive models, including Random Forest (RF), Light Gradient Boosting Machine (LGBM), eXtreme Gradient Boosting (XGB), Decision Tree (DT), K-Nearest Neighbors (KNN), Multi-Layer Perceptron (MLP), AdaBoost (AB), Support Vector Machine (SVM), Logistic Regression (LR), and Naive Bayes Classifier (NBC). All preprocessing steps, including KNN imputation, were performed exclusively on the training set. Hyperparameter tuning was performed using random search with 10-fold cross-validation on the training set only. No scaling was applied because tree-based models are invariant to monotonic transformations. Feature selection via logistic regression was conducted on the training set alone; the same selected features were used across all cross-validation folds. During the model development phase, hyperparameters were optimized using random search combined with 10-fold cross-validation.

The final Random Forest model is specified as follows. The model uses seven predictors: dyspnea, serum ferritin, wheezing, immunoglobulin G, interleukin-6, preterm birth, and personal history of wheezing. Hyperparameters were set to: number of trees = 500, maximum tree depth = 30, minimum samples required to split an internal node = 2, minimum samples required at a leaf node = 1. Bootstrap sampling was used to train each tree, and at each split a random subset of features was considered [default sqrt(number of features) in scikit-learn]. The predicted probability of PICU admission for a patient is the average of the predicted probabilities across all 500 trees. A patient is classified as high risk (i.e., predicted to require PICU admission) if this probability is ≥0.5 (default threshold). Subsequently, a second round of 10-fold cross-validation was performed to systematically evaluate the performance of each model. The evaluation metrics included AUROC, AP, Accuracy, Precision, Recall, and F1 Score to comprehensively assess the model’s discriminative ability and robustness. Based on the results of cross-validation, the model with the highest AUROC was selected as the final candidate model. The selected model was further evaluated in the internal test set and temporal external validation cohort, and calibration curves were used to evaluate the consistency between predicted probabilities and actual outcomes. Finally, Decision Curve Analysis (DCA) was employed to quantify the net clinical benefit of the model at various decision thresholds, thereby assessing its potential clinical utility across different threshold probabilities.

### Statistical analysis

All analyses were performed in R 4.2.3 (R Foundation for Statistical Computing) and Python 3.8 (Python Software Foundation). Initially, the Shapiro-Wilk test was conducted to assess the normality of continuous variables. For normally distributed variables, data are presented as mean ± standard deviation, and intergroup comparisons were made using the independent *t*-test. For non-normally distributed variables, data are presented as median (interquartile range), and comparisons were performed using the Mann-Whitney U test. Categorical variables are presented as frequencies (percentages), with group comparisons conducted using the χ^2^ test or Fisher’s exact test.

To identify predictors of PICU admission (yes/no), univariable logistic regression was conducted for all candidate variables, with variables showing *P* < 0.10 being included in the multivariable logistic regression model. Univariable and multivariable logistic regression for feature selection were performed only on the training set. Although feature selection was not nested within each cross-validation fold, the selected predictors were clinically stable and based solely on training data, minimizing the risk of data leakage. Logistic regression-based feature screening was used to develop a parsimonious and clinically interpretable model and to reduce the risk of overfitting. However, this approach may not fully capture predictors with non-linear or interaction effects.

A backward elimination approach was used in the multivariable logistic regression model to identify predictors retained for machine learning model development, and the results are presented as odds ratios (ORs) with 95% confidence intervals (CIs). A two-tailed *P*-value < 0.05 was considered statistically significant. In the model development and evaluation phase, the significant features selected from the regression analysis were incorporated into the machine learning feature set for further analysis. All statistical analyses were performed using standard methods in R and Python, ensuring the reliability of the data processing and results.

Using the pmsampsize framework (21), the development cohort (1,606 patients, 209 events) meets requirements for developing a prediction model with expected AUROC of 0.85. The final model has seven predictors, giving EPV = 209/7 ≈ 29.9, exceeding the recommended minimum of 10–20.

### Code availability

The Python and R scripts used for data preprocessing, missing data imputation, model training, hyperparameter tuning, performance evaluation, and SHAP analysis are available from the corresponding author upon reasonable request.

### Patient and public involvement

Patients and the public were not involved in the design, conduct, reporting, or dissemination of this retrospective study.

### Sensitivity analysis

To address the limited availability of certain biomarkers in resource-constrained settings and to assess the practical applicability of the model, we conducted a sensitivity analysis using only routinely available clinical variables and laboratory tests. Based on this reduced feature set, we retrained the model and evaluated its performance in both the internal test set and the temporal external validation cohort.

## Results

Between 1 January 2023 and 31 March 2025, 1,892 hospitalized pediatric patients aged 29 days to 18 years with confirmed RSV infection were screened. After exclusions, 68 patients with severe underlying conditions, 94 with missing key clinical data, and 124 with incomplete laboratory parameters were omitted from the analysis. As a result, 1,606 patients were included in the final analysis, of whom 209 were transferred to the PICU and 1,397 were managed in the general pediatric ward (Supplementary Figure 1). The training set contained 1,285 patients (167 PICU admissions, 13.0%), the internal test set 321 patients (42 PICU admissions, 13.0%), and the temporal external validation cohort 94 patients (12 PICU admissions, 12.8%), (Supplementary Table 1). The age range of patients in the general pediatric ward was from 1.00 to 177.00 months, while the age range in the pediatric intensive care unit was from 1.00 to 156.00 months. The baseline characteristics of the two groups are presented in Table 1. For validation, we analyzed data from another cohort of children admitted between 1 April and 30 April 2025. The children in the PICU group were significantly younger than those in the general pediatric ward group (median age 11.84 months [IQR, 5.23–15.09] vs. 20.96 months [IQR, 12.00–22.74]; *P* < 0.001), and the proportion of preterm infants was notably higher in the PICU group (57/209 [27.3%] vs. 45/1,397 [3.2%]; *P* < 0.001). Although no significant differences were observed between the two groups in terms of gender and history of allergic diseases, a higher proportion of patients in the PICU group had a history of wheezing (41/209 [19.6%] vs. 106/1,397 [7.6%], *P* < 0.001). Additionally, the proportion of breastfeeding was lower in the PICU group (149/209 [71.3%] vs. 1,213/1,397 [86.8%], *P* < 0.001). In terms of clinical manifestations, the PICU group exhibited more severe symptoms compared to the general pediatric ward. While the incidence of fever and cough was similar between the groups, the PICU group showed a significantly higher rate of wheezing (159/209 [76.1]% vs. 277/1,397 [19.8%]) and an even more pronounced increase in the incidence of dyspnea (116/209 [55.5%] vs. 2/1,397 [0.1%]). On the first day of hospitalization, the PICU group had significantly higher levels of white blood cell count, neutrophil count, platelet, procalcitonin, serum ferritin, and interleukin-6, while their hemoglobin and immunoglobulin G levels were significantly lower (all *P* < 0.001). Missing data were uncommon among the candidate predictors, and the complete missing data summary is provided in Supplementary Table 4. The highest missing rate was observed for white blood cell count, with 34 missing values (2.1%). Missing rates were 0.7% for preterm birth, 1.2% for personal history of atopy, 0.7% for personal history of wheezing, and 1.1% for breastfeeding. Most laboratory indicators had no missing values. Key biomarkers included in the final model, including interleukin-6, serum ferritin, and immunoglobulin G, were available for all included patients, with missing rates of 0%.

**TABLE 1:** Baseline demographic and clinical characteristics of the study patients.

Characteristics	Overall (1,606)	GPW (1,397)	PICU (209)	*P*
Demographic characteristics				
Age, months, median (IQR)	19.78 (9.20, 23.50)	20.96 (12.00, 22.74)	11.84 (5.23, 15.09)	<0.001
Male, *n* (%)	611 (38.0)	538 (38.5)	73 (34.9)	0.36
Medical history, n (%)				
Preterm birth	102 (6.4)	45 (3.2)	57 (27.3)	<0.001
Personal history of atopy	161 (10.0)	136 (9.7)	25 (12.0)	0.38
Personal history of wheezing	147 (9.2)	106 (7.6)	41 (19.6)	<0.001
Breast feeding	1,362 (84.8)	1,213 (86.8)	149 (71.3)	<0.001
Clinical characteristics, n (%)				
Fever	1,029 (64.1)	905 (64.8)	124 (59.3)	0.02
Cough	1,604 (99.9)	1,395 (99.9)	209 (100)	0.91
Wheezing	436 (27.1)	277 (19.8)	159 (76.1)	<0.001
Dyspnea	118 (7.3)	2 (0.1)	116 (55.5)	<0.001
Laboratory indices, mean (SD)				
White blood cell count, ×10^9^/L	9.77 (4.73)	9.58 (3.88)	11.08 (8.35)	<0.001
Absolute neutrophil count, ×10^9^/L	3.98 (3.38)	3.82 (3.24)	5.03 (4.04)	<0.001
Absolute lymphocyte count, ×10^9^/L	5.30 (4.45)	5.40 (4.52)	4.62 (3.82)	0.02
Hemoglobin, g/L	113.37 (26.99)	114.28 (28.26)	107.31 (14.77)	<0.001
Platelet, ×10^9^/L	380.35 (152.38)	370.72 (104.44)	444.72 (318.08)	<0.001
C-reactive protein, mg/L	6.96 (16.64)	6.46 (15.05)	10.27 (24.54)	0.002
Procalcitonin, ng/mL	0.37 (2.38)	0.29 (1.96)	0.95 (4.17)	<0.001
Serum ferritin, ng/mL	85.12 (107.16)	73.78 (77.97)	160.94 (202.91)	<0.001
Interleukin-6, pg/mL	26.19 (123.90)	19.23 (39.65)	72.70 (324.67)	<0.001
Alanine aminotransferase, U/L	28.42 (28.41)	27.54 (25.16)	34.31 (44.02)	0.001
Aspartate aminotransferase, U/L	49.19 (95.14)	49.00 (101.12)	50.43 (34.77)	0.02
Creatine kinase, U/L	113.46 (95.01)	113.33 (92.45)	114.32 (110.88)	0.89
Creatine kinase myocardial band, U/L	30.31 (46.98)	29.85 (37.36)	33.36 (87.47)	0.31
Lactate dehydrogenase, U/L	310.03 (155.77)	308.44 (162.97)	320.62 (93.99)	0.29
Urea, mmol/L	3.60 (11.42)	3.54 (10.74)	4.01 (15.23)	0.58
Creatinine, μmol/L	25.02 (27.39)	25.27 (27.98)	23.37 (23.11)	0.35
Uric acid, μmol/L	244.35 (78.74)	244.69 (76.77)	242.08 (90.99)	0.66
Sodium, mmol/L	140.41 (4.28)	142.34 (5.36)	138.38 (6.58)	0.72
Calcium, mmol/L	2.62 (7.21)	2.57 (6.81)	3.00 (9.48)	0.42
Prothrombin time, s	11.90 (4.83)	11.91 (5.13)	11.83 (1.79)	0.82
Activated partial thromboplastin time, s	31.15 (11.41)	31.06 (11.80)	31.80 (8.35)	0.38
D-dimer, μg/mL	0.58 (3.15)	0.50 (3.22)	1.09 (2.56)	0.01
Antithrombin III, %	103.94 (14.48)	104.39 (13.86)	100.93 (17.84)	0.001
Immunoglobulin A, g/L	0.69 (2.28)	0.73 (2.43)	0.39 (0.44)	0.04
Immunoglobulin M, g/L	0.95 (0.62)	0.96 (0.57)	0.90 (0.86)	0.21
Immunoglobulin G, g/L	5.38 (2.06)	5.50 (1.71)	4.58 (3.50)	<0.001

GPW, general pediatric ward; PICU, pediatric intensive care unit; IQR, interquartile range; SD, standard deviation.

The distribution of RSV diagnostic methods is shown in Supplementary Table 5. Among the included patients, 1,284 children were confirmed by nucleic acid testing and 322 by rapid antigen testing. In the general pediatric ward group, 1,110/1,397 patients (79.5%) were confirmed by nucleic acid testing and 287/1,397 (20.5%) by rapid antigen testing. In the PICU group, 174/209 patients (83.3%) were confirmed by nucleic acid testing and 35/209 (16.7%) by rapid antigen testing. The distribution of testing methods was not significantly different between the two groups (*P* = 0.201).

We performed univariable and multivariable logistic regression analyses on the training set data to select variables for inclusion in the machine learning model. The univariable analysis identified 21 variables significantly associated with PICU admission (Table 2), including age, preterm birth, personal history of wheezing, breast feeding, fever, cough, wheezing, dyspnea, white blood cell count, absolute neutrophil count, absolute lymphocyte count, platelet, hemoglobin, c-reactive protein, procalcitonin, serum ferritin, interleukin-6, alanine aminotransferase, antithrombin III, immunoglobulin A, immunoglobulin G. In the multivariable logistic regression model, preterm birth, personal history of wheezing, wheezing, dyspnea, serum ferritin, interleukin-6, and immunoglobulin G were retained as predictors of PICU admission (Table 2).

**TABLE 2:** Univariable and multivariable logistic regression analysis of predictors for PICU admission.

Variable	Univariable analysis		Multivariable analysis	
	Univariable OR (95% CI)	*P*	Multivariable OR (95% CI)	*P*
Age, months	0.97 (0.96–0.98)	*p* < 0.001*	0.98 (0.95–1.01)	*p* = 0.17
Male	0.86 (0.64–1.17)	*p* = 0.35	NA	NA
Preterm birth	11.33 (7.41–17.34)	*p* < 0.001*	4.79 (1.50–15.25)	*p* = 0.008
Personal history of atopy	1.27 (0.80–1.99)	*p* = 0.31	NA	NA
Personal history of wheezing	3.02 (2.03–4.48)	*p* < 0.001*	1.64 (1.20–1.93)	*p* = 0.001
Breast feeding	0.38 (0.27–0.53)	*p* < 0.001*	1.67 (0.56–5.01)	*p* = 0.36
Fever	1.07 (1.01–1.14)	*p* = 0.02	0.98 (0.82–1.17)	*p* = 0.83
Cough	1.10 (1.06–1.15)	*p* < 0.001*	0.92 (0.80–1.07)	*p* = 0.29
Wheezing	1.52 (1.37–1.68)	*p* < 0.001*	1.22 (1.14–1.30)	*p* = 0.02
Dyspnea	878.83 (213.84–3611.80)	*p* < 0.001*	837.44 (100.73–6942.43)	*p* < 0.001*
White blood cell count, ×10^9^/L	1.06 (1.03–1.09)	*p* < 0.001*	1.01 (0.90–1.13)	*p* = 0.85
Absolute neutrophil count, × 10^9^/L	1.08 (1.04–1.13)	*p* < 0.001*	1.07 (0.95–1.20)	*p* = 0.29
Absolute lymphocyte count, × 10^9^/L	0.92 (0.87–0.97)	*p* = 0.004	0.93 (0.80–1.08)	*p* = 0.35
Hemoglobin, g/L	0.96 (0.95–0.98)	*p* < 0.001*	1.00 (0.99–1.02)	*p* = 0.62
Platelet, ×10^9^/L	1.00 (1.00–1.01)	*p* < 0.001*	1.00 (0.99–1.00)	*p* = 0.23
C-reactive protein, mg/L	1.01 (1.00–1.02)	*p* = 0.008	1.01 (0.99–1.03)	*p* = 0.43
Procalcitonin, ng/mL	1.07 (1.02–1.13)	*p* = 0.008	1.01 (0.77–1.32)	*p* = 0.95
Serum ferritin, ng/mL	1.01 (1.01–1.01)	*p* < 0.001*	1.00 (1.00–1.01)	*p* = 0.004
Interleukin-6, pg/mL	1.01 (1.00–1.01)	*p* < 0.001*	1.00 (1.00–1.01)	*p* = 0.003
Alanine aminotransferase, U/L	1.01 (1.00–1.01)	*p* = 0.003	1.00 (0.99–1.01)	*p* = 0.63
Aspartate aminotransferase, U/L	1.00 (1.00–1.00)	*p* = 0.84	NA	NA
Creatine kinase, U/L	1.00 (1.00–1.00)	*p* = 0.88	NA	NA
Creatine kinase myocardial band, U/L	1.00 (1.00–1.00)	*p* = 0.35	NA	NA
Lactate dehydrogenase, U/L	1.00 (1.00–1.00)	*p* = 0.31	NA	NA
Urea, mmol/L	1.00 (0.99–1.01)	*p* = 0.58	NA	NA
Creatinine, μmol/L	0.99 (0.98–1.01)	*p* = 0.34	NA	NA
Uric acid, μmol/L	1.00 (1.00–1.00)	*p* = 0.67	NA	NA
Sodium, mmol/L	1.00 (1.00–1.00)	*p* = 0.79	NA	NA
Calcium, mmol/L	1.01 (0.99–1.02)	*p* = 0.45	NA	NA
Prothrombin time, s	1.00 (0.96–1.03)	*p* = 0.83	NA	NA
Activated partial thromboplastin time, s	1.00 (0.99–1.01)	*p* = 0.38	NA	NA
D-dimer, μg/mL	1.04 (0.99–1.10)	*p* = 0.16	NA	NA
Antithrombin III, %	0.98 (0.97–0.99)	*p* = 0.001*	1.01 (0.98–1.03)	*p* = 0.67
Immunoglobulin A, g/L	0.05 (0.02–0.08)	*p* < 0.001	0.31 (0.10–1.96)	*p* = 0.71
Immunoglobulin M, g/L	0.82 (0.60–1.13)	*p* = 0.23	NA	NA
Immunoglobulin G, g/L	0.71 (0.64–0.79)	*p* < 0.001*	0.70 (0.57–0.86)	*p* < 0.001*

PICU, pediatric intensive care unit; OR, odds ratio; CI, confidence interval; NA, not applicable. *P < 0.05.

Univariable performance of dyspnea alone. Because dyspnea was the strongest predictor and overlaps with PICU admission criteria, we evaluated its performance as a single binary predictor in the internal test set. Dyspnea alone achieved an AUROC of 0.77, sensitivity (recall) of 0.55, specificity of 0.996, precision of 0.96, and F1 score of 0.70. Detailed metrics are provided in Supplementary Table 6.

**FIGURE 1:**
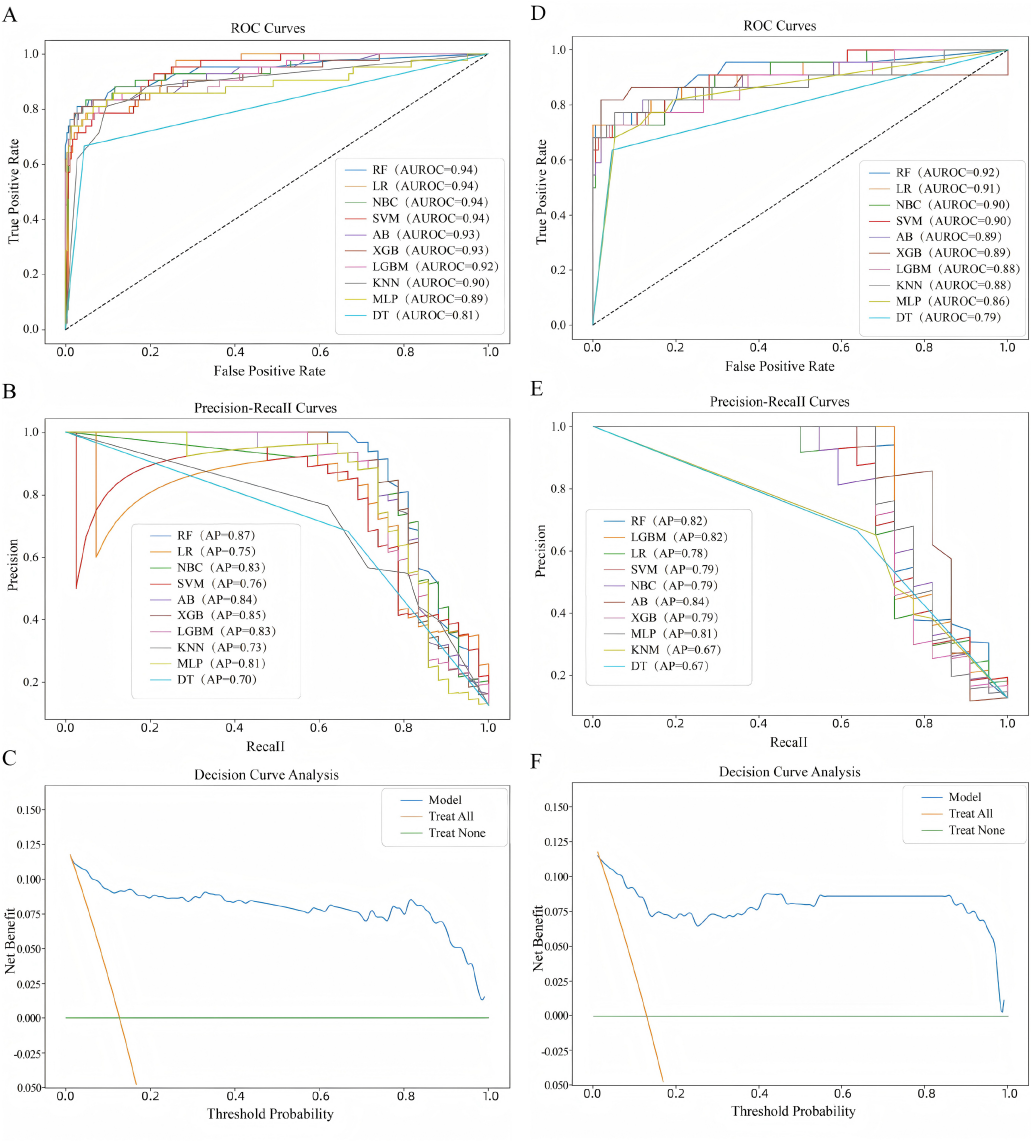
ROC curves of 10 machine learning models in the internal test set **(A)**, PR curves of ten machine learning models in the internal test set **(B)**, and DCA curves of RF machine learning models in the internal test set **(C)**. ROC, PR, and DCA curves of 10 machine learning models in the temporal external validation cohort. **(D)** ROC, **(E)** PR, and **(F)** DCA curves.

The hyperparameter search space for each machine learning algorithm is provided in Supplementary Table 3. The ROC, PR, and DCA curves of the ten models in the internal test set and temporal external validation cohort are presented in Figure 1. The performance of the ten models in the training set, internal test set, and temporal external validation cohort is summarized in Supplementary Table 1. Overall, the RF model showed the best overall performance among the ten models.

In the internal test set, where the prevalence of PICU admission was 13.0%, the RF model achieved an AUROC of 0.94, an AP of 0.87, an accuracy of 0.95, a precision of 0.86, a recall of 0.76, and an F1 score of 0.81. The AP of 0.87 was substantially higher than the random baseline AP of 0.13. In the temporal external validation cohort, where the prevalence of PICU admission was 12.8%, the RF model achieved an AUROC of 0.92, an AP of 0.82, an accuracy of 0.95, a precision of 0.94, a recall of 0.68, and an F1 score of 0.79. The AP of 0.82 likewise demonstrated considerable improvement over the random baseline AP of 0.128. Calibration curves for the ten models across the training, internal test, and temporal external validation sets are shown in Supplementary Figure 2.

Threshold and subgroup performance. The default classification threshold was 0.5. Subgroup analyses were performed to assess model consistency across clinically relevant strata. In infants aged < 12 months (*n* = 833), the RF model achieved an AUROC of 0.91, recall of 0.73, and F1 of 0.79; in children ≥ 12 months (*n* = 773), AUROC was 0.93, recall 0.78, and F1 0.82. By preterm birth status, the model showed an AUROC of 0.89 in preterm infants (*n* = 102) and 0.94 in term children (*n* = 1,504), with corresponding recall of 0.67 and 0.79, and F1 of 0.71 and 0.83, respectively. Detailed subgroup performance is presented in Supplementary Table 7.

To assess whether the model merely recapitulates clinical criteria, we retrained the Random Forest model after excluding dyspnea and wheezing (the two clinical signs most directly overlapping with PICU admission criteria). Using the remaining predictors (serum ferritin, immunoglobulin G, interleukin-6, preterm birth, personal history of wheezing, and other non-overlapping clinical and laboratory variables), the reduced model achieved an AUROC of 0.87, recall of 0.68, precision of 0.77, and F1 of 0.71 in the internal test set. In the temporal external validation cohort, the reduced model achieved an AUROC of 0.84, recall of 0.62, precision of 0.72, and F1 of 0.67. A full comparison is shown in Supplementary Table 6.

We conducted a sensitivity analysis using only routinely available clinical data. Based on this subset of variables, the RF model achieved an AUROC of 0.89 in the internal test set and 0.87 in the temporal external validation cohort, with a recall of 0.71 and an F1 score of 0.75. These results suggest that the model retained predictive performance when limited to commonly measured variables, although further external validation is needed before clinical application (Supplementary Table 2).

**FIGURE 2:**
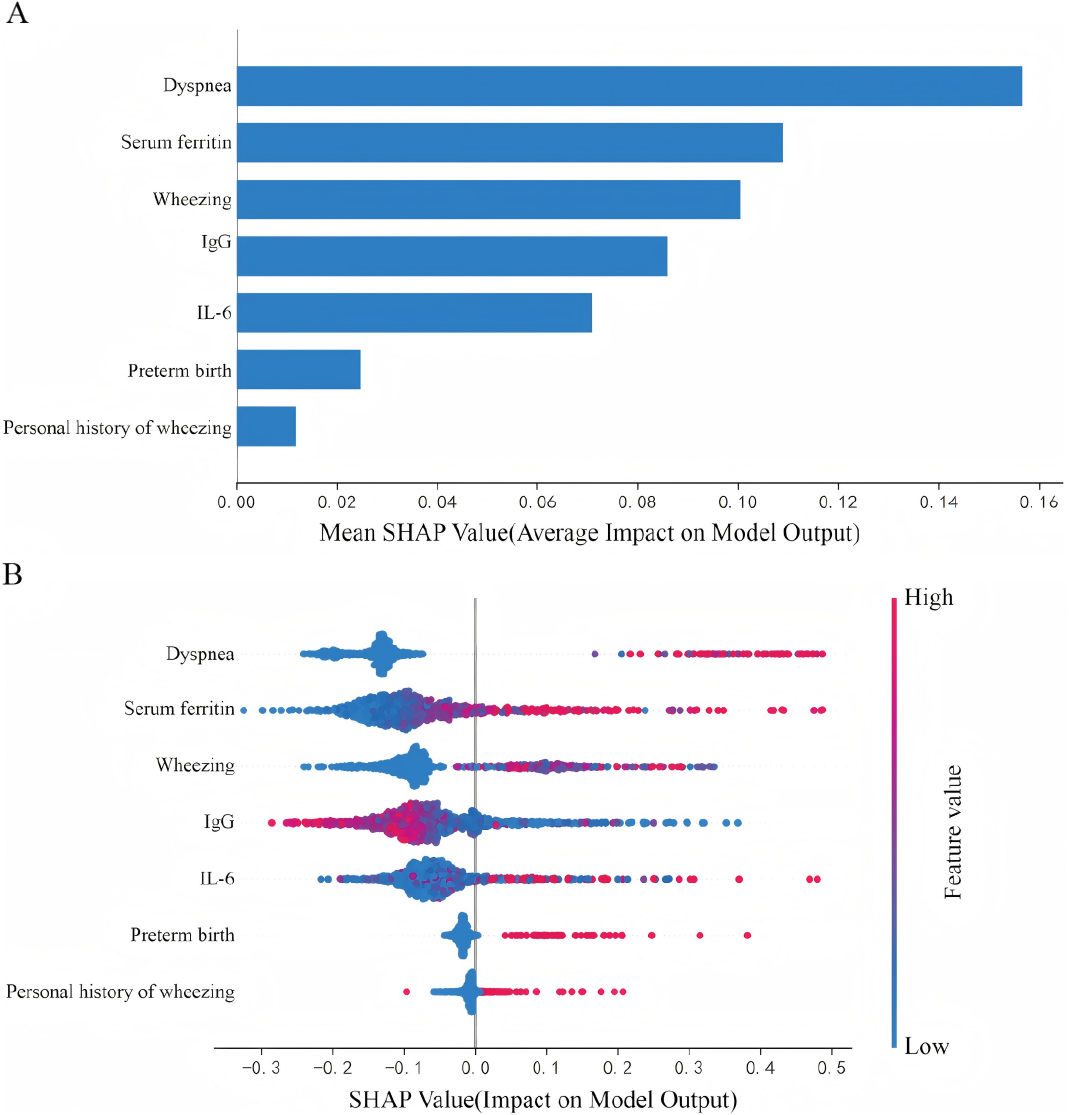
Relative importance of variables based on SHAP for the RF prediction model. **(A)** Mean absolute SHAP values identifying key predictors of PICU admission: dyspnea, serum ferritin, wheezing, immunoglobulin G, interleukin-6, preterm birth, and personal history of wheezing. **(B)** Scatter plot displays SHAP values for the same features. each dot represents an individual patient, and color indicates feature value from low (blue) to high (pink).

**FIGURE 3:**
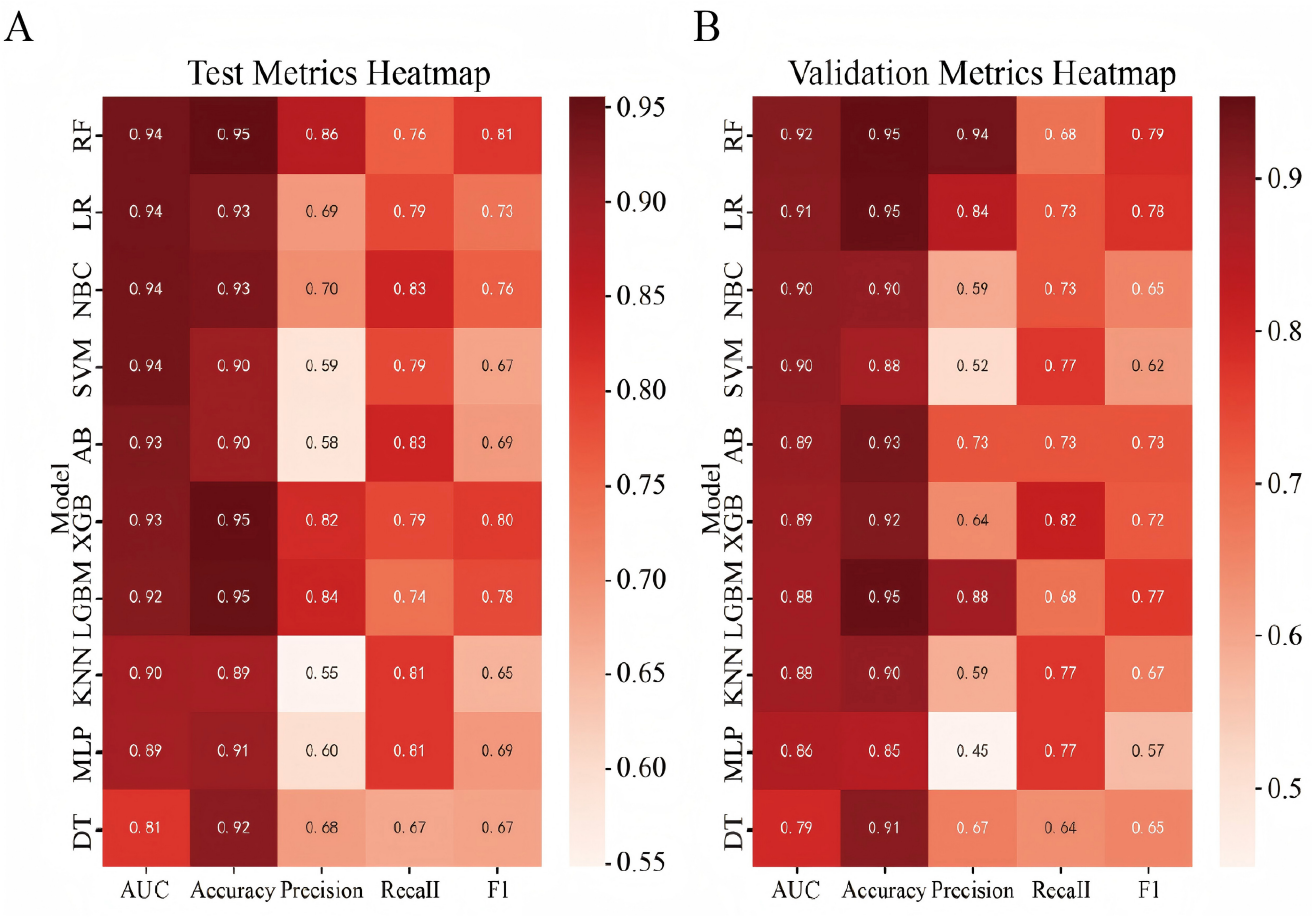
Comparison of model performance across internal test **(A)** and temporal external validation **(B)** cohorts: a heatmap analysis of AUC, accuracy, precision, recall, and F1 score.

The global feature importance ranking based on SHAP values (Figure 2A) indicates that dyspnea, serum ferritin, wheezing, immunoglobulin G, interleukin-6, preterm birth, and personal history of wheezing were the seven most influential features, with mean SHAP values of 0.16, 0.11, 0.10, 0.09, 0.07, 0.02, and 0.01, respectively. The individual scatter plot (Figure 2B) further demonstrates that the presence of dyspnea, wheezing, preterm birth, a personal history of wheezing, elevated serum ferritin levels, increased interleukin-6 levels, and low immunoglobulin G concentrations were significantly associated with an increased risk of PICU admission. The model performance heatmap (Figure 3) provides a visual comparison of discrimination and threshold-dependent classification metrics across the ten algorithms in the internal test set and temporal external validation cohort. The RF model was selected as the final model because it showed the most balanced overall performance, rather than superiority in a single metric alone. In the internal test set, the RF model achieved high AUROC and AP, together with favorable accuracy, precision, recall, and F1 score. This performance pattern was generally maintained in the temporal external validation cohort, although recall decreased to 0.68. This lower recall indicates that some children who were subsequently transferred to the PICU may not have been classified as high risk at the selected threshold. Therefore, while Figure 3 supports the RF model as the best overall candidate among the compared algorithms, the trade-off between precision and recall should be considered when applying the model to PICU triage.

## Discussion

This study developed an interpretable machine learning model to predict actual PICU transfer among children hospitalized with RSV infection. Among the ten commonly used algorithms compared in this study, the RF model showed relatively favorable performance in the internal test set, with an AUROC of 0.94, AP of 0.87, accuracy of 0.95, precision of 0.86, recall of 0.76, and F1 score of 0.81. SHAP analysis identified dyspnea, serum ferritin, wheezing, immunoglobulin G, interleukin-6, preterm birth, and personal history of wheezing as important contributors to model predictions. These predictors are clinically plausible and may reflect respiratory compromise, inflammatory response, immune status, and underlying vulnerability in children with RSV infection.

Previous study (9) have primarily relied on perinatal or early postnatal factors—such as delivery conditions and congenital anomalies—to predict the likelihood of severe illness and ICU admission in infants who may develop RSV infection in the future. These models have been mainly applied at the population level for risk stratification and preventive strategies, such as determining eligibility for monoclonal antibody administration or prioritizing vaccination. In contrast, the present study focuses on pediatric patients already infected with RSV. Using clinical information and blood biomarkers available at the time of hospital presentation, we developed a model to predict PICU admission. This approach aims to enable earlier identification of high-risk children and to support more precise clinical interventions. The RF model constructed in this study showed favorable performance in the internal test set, achieving an area under the AUROC of 0.94, an AP of 0.87, an accuracy of 0.95, a precision of 0.86, a recall of 0.76, and an F1 score of 0.81. In the temporal external validation cohort, the RF model showed relatively favorable performance, with an AUROC of 0.92, an AP of 0.82, an accuracy of 0.95, a precision of 0.94, a recall of 0.68, and an F1 score of 0.79.

Decision curve analysis and calibration plots suggested potential clinical utility across a range of threshold probabilities and acceptable calibration between predicted probabilities and observed outcomes. SHAP-based interpretation identified key predictors of PICU admission, including dyspnea, serum ferritin, preterm birth, wheezing, immunoglobulin G, interleukin-6, and a personal history of wheezing. Several of these variables are consistent with previously reported (22) high-risk factors, such as prematurity and comorbidities. Unlike earlier study (22) that relied primarily on static demographic data and the binary presence of comorbidities, our study provided a more nuanced classification of comorbidity types and incorporated dynamic biomarkers indicative of inflammatory and immune responses, such as serum ferritin, immunoglobulin G, and IL-6. By integrating a machine learning model with SHAP-based interpretation, our approach may improve model interpretability and support early risk stratification. This multidimensional modeling strategy may enhance potential clinical applicability after further validation, particularly in resource-limited settings, by supporting more precise patient triage and optimized resource allocation.

In practice, the model may be integrated into the electronic health record system to provide early risk scores after hospital admission and support clinician awareness of children at higher risk of subsequent PICU transfer. However, the recall of 0.68 in the temporal external validation cohort indicates that approximately one-third of children who were subsequently transferred to the PICU were not identified as high risk at the selected classification threshold. This is clinically important because false-negative predictions may delay escalation of monitoring or critical care support. Therefore, the model should not be used as a stand-alone triage tool. If prospectively validated, a lower decision threshold could improve sensitivity and reduce missed high-risk patients, but at the cost of more false-positive alerts and potentially unnecessary intensive monitoring or PICU evaluation. Moreover, the model’s interpretability via SHAP may help clinicians understand the key contributing factors for each patient, such as dyspnea or elevated serum ferritin levels, thereby supporting individualized risk assessment. Future prospective studies should evaluate threshold selection according to clinical priorities, patient safety considerations, and local resource availability.

Although this study developed an interpretable model for predicting PICU admission in children with RSV infection, several limitations should be acknowledged. First, the single-center retrospective design limits generalizability. All data came from one tertiary maternal and child health hospital in Luoyang, China, and local patient characteristics, PICU admission thresholds, workflows, and resource availability may not reflect other settings. Although the model was assessed using an 80/20 split-sample internal test set, 10-fold cross-validation within the training set, and a temporal external validation cohort of newly accrued patients from April 2025 (*n* = 94, 12 events), this temporal external validation, while a valid form of external validation per TRIPOD/TRIPOD-AI methodology, has limited precision due to its small sample size. It does not, however, constitute geographic, multicenter, or healthcare-system external validation. Because bootstrap-based optimism correction was not performed, performance estimates from model development may remain optimistic. In addition, the temporal external validation cohort included few events, limiting the precision of validation estimates. Larger multicenter external validation across diverse populations, healthcare systems, and RSV epidemic phases is required before clinical implementation. Second, although the final model included seven predictors and the development cohort contained 209 outcome events, yielding an events-per-variable ratio of approximately 29.9, this calculation applies only to the final selected model and does not fully capture the complexity of the overall model-development process. The initial candidate predictor set was larger, and logistic regression-based feature screening was used to reduce dimensionality. Although this strategy was intended to improve parsimony, it does not eliminate the risk of overfitting, particularly because feature selection was not nested within each cross-validation fold and multiple machine learning algorithms were compared. Moreover, bootstrap-based internal validation was not performed; therefore, the reported discrimination and calibration were not optimism-corrected and may be overestimated. Future studies with larger numbers of outcome events, prespecified sample size calculations, and bootstrap-based internal validation are needed to obtain more reliable performance estimates. Third, RSV infection was confirmed by either nucleic acid or rapid antigen testing, reflecting routine clinical practice but introducing potential diagnostic ascertainment or work-up bias. The retrospective dataset did not allow reliable retraining and validation restricted to nucleic acid-confirmed cases. We also lacked virological and host-level data, including viral load, RSV subtype, and host genetic information, which might further improve risk prediction but are not routinely available in many clinical settings. In addition, although interleukin-6, serum ferritin, and immunoglobulin G were available in the analytical cohort, these biomarkers may not be routinely measured in resource-limited settings, potentially restricting model transportability. Our sensitivity analysis using routinely available variables partly addressed this concern, but future studies should use standardized RSV testing, uniform case definitions, and simplified models based on universally accessible predictors. Fourth, personal history of wheezing was derived from retrospective electronic health records and coded as a binary variable. More detailed and reproducible measures, including physician-diagnosed recurrent wheeze, frequency of previous wheezing episodes, and prior hospitalization for wheezing or respiratory distress, were unavailable. Standardized definitions should be incorporated in future prospective studies to improve predictor reliability and model transportability. Fifth, some predictors, particularly dyspnea and wheezing, overlap with clinical criteria for PICU transfer, raising the possibility of incorporation bias and overestimated performance. In a reduced model excluding these variables, discrimination remained acceptable (AUROC 0.87), suggesting that other clinical and laboratory predictors contributed additional information. Nevertheless, because the outcome was actual PICU transfer recorded in electronic health records rather than an independently adjudicated need for intensive care, blinded outcome assessment was not feasible. This may have introduced outcome assessment bias, especially for predictors closely linked to transfer decisions. Future prospective studies should use independent outcome adjudication where possible and evaluate the model in patients for whom the need for PICU care is clinically uncertain. Sixth, although multiple performance metrics were reported in the internal test and temporal external validation cohort, 95% confidence intervals, formal calibration intercept/slope, Brier score, specificity, and NPV were not estimated in the current analysis. Future external validation studies should report interval estimates, calibration statistics, and complete threshold-dependent performance metrics to more fully quantify the uncertainty, calibration, and clinical applicability of model performance. Seventh, the main model used logistic regression-based feature screening before machine learning model development. Although this strategy was intended to develop a parsimonious and clinically interpretable model and reduce overfitting, *P*-value-based screening may remove predictors with non-linear or interaction effects. In addition, although feature selection was performed only on the training set to reduce data leakage, it was not nested within each cross-validation fold, which may have introduced some risk of over-optimistic performance estimates. Future studies should compare this approach with models using all clinically prespecified predictors, regularization-based feature selection, embedded feature selection methods within machine learning algorithms, or nested cross-validation frameworks. Eighth, we did not formally assess algorithmic fairness across sex, socioeconomic status, or race/ethnicity because the study was conducted in a single center with a largely homogeneous population. Future external validation should include fairness evaluation in diverse settings. Ninth, the study was not prospectively registered; this is a limitation inherent to retrospective prediction model studies and has been noted.

## Conclusion

We developed an interpretable machine learning model that predicted PICU transfer in children with RSV infection. The random forest model showed favorable performance and SHAP analysis improved interpretability, suggesting potential for early risk stratification. Nevertheless, development estimates may remain optimistic without bootstrap-based optimism correction, and the small temporal external validation cohort limits precision. Further geographical, institutional, and healthcare-system external validation is required before clinical implementation.

## Data Availability

The raw data supporting the conclusions of this article will be made available by the authors, without undue reservation.
