# Supplementary material for: Risk prediction of pediatric intensive care unit admission in children with respiratory syncytial virus infection using interpretable machine learning
Source: Front Med (Lausanne). 2026 Jul 8;13:1856930. doi: 10.3389/fmed.2026.1856930 (PMC13388268; doi:10.3389/fmed.2026.1856930)

**Supplement Information**

| **Table S1.** Performance Comparison of Ten Machine Learning Models in the Training Set, Internal Test Set, and Short Time-Based External Validation Cohort. |
| --- |
| **Table S2.** Sensitivity Analysis Using Routinely Available Clinical Variables and Laboratory Tests in the Internal Test Set and Short Time-Based External Validation Cohort. |
| **Table S3.** Hyperparameter Search Space for Machine Learning Models. |
| **Table S4.** Missing Data Summary for Predictor Variables. |
| **Table S5.** Distribution of RSV Diagnostic Methods Stratified by Ward Location. |
| **Table S6.** Univariable performance of dyspnea alone and performance of the Random Forest model after excluding predictors overlapping with PICU admission criteria. |
| **Table S7.** Subgroup performance of the Random Forest model for predicting PICU admission. |
| **Fig S1.** Flow chart of enrollment. |
| **Fig S2.** Performance Comparison of Ten Machine Learning Models During Training: Calibration Curves for theTraining Set, Internal Test Set, and Short Time-Based External Validation Cohort. |

**Table S1.** Performance Comparison of Ten Machine Learning Models in the Training Set, Internal Test Set, and Short Time-Based External Validation Cohort.

|  | **Model** | **AUC**  **（%）** | **AP**  **（%）** | **Accuracy**  **（%）** | **Precision**  **（%）** | **Recall**  **（%）** | **F1**  **（%）** |
| --- | --- | --- | --- | --- | --- | --- | --- |
| **Training set (N=1,285, PICU events=167)** | RF | 98.5 | 96.9 | 96.8 | 96.3 | 96.2 | 96.4 |
|  | LGBM | 97.8 | 96.5 | 96.6 | 93.7 | 97.1 | 95.3 |
|  | XGB | 96.7 | 96.9 | 96.4 | 96.1 | 96.1 | 96.3 |
|  | DT | 96.6 | 96.5 | 96.5 | 96.3 | 96.2 | 96.3 |
|  | KNN | 96.3 | 94.1 | 90.9 | 58.9 | 97.9 | 73.6 |
|  | MLP | 94.7 | 92.5 | 93.2 | 67.9 | 90.3 | 77.5 |
|  | AB | 93.5 | 81.8 | 88.2 | 52.9 | 81.4 | 64.1 |
|  | SVM | 92.6 | 77.5 | 88.7 | 54.1 | 82.8 | 65.4 |
|  | LR | 91.9 | 77.2 | 89.1 | 55.8 | 75.9 | 64.3 |
|  | NBC | 87.4 | 70.6 | 89.9 | 60.5 | 63.4 | 62.0 |
| **Internal test set**  **(N=321, PICU events=42)** | RF | 94.1 | 87.2 | 95.3 | 86.4 | 76.2 | 81.0 |
|  | LR | 94.1 | 74.7 | 92.8 | 68.8 | 78.6 | 73.3 |
|  | NBC | 94.0 | 83.1 | 93.4 | 70.0 | 83.3 | 76.1 |
|  | SVM | 94.0 | 75.5 | 90.4 | 58.9 | 78.6 | 67.3 |
|  | AB | 93.0 | 84.0 | 90.4 | 58.3 | 83.3 | 68.6 |
|  | XGB | 93.1 | 84.7 | 95.2 | 82.5 | 78.6 | 80.5 |
|  | LGBM | 92.4 | 83.0 | 94.9 | 83.8 | 73.8 | 78.4 |
|  | KNN | 90.1 | 72.6 | 89.2 | 54.8 | 81.0 | 65.4 |
|  | MLP | 89.4 | 81.0 | 90.7 | 59.6 | 81.0 | 68.7 |
|  | DT | 81.1 | 69.6 | 91.9 | 68.3 | 66.7 | 67.2 |
| **Short time‑based external validation cohort**  **(N=94, PICU events=12)** | RF | 92.4 | 82.0 | 95.3 | 94.4 | 68.1 | 79.1 |
|  | LGBM | 88.2 | 81.6 | 94.8 | 84.2 | 72.7 | 78.0 |
|  | LR | 90.6 | 78.1 | 90.1 | 59.3 | 72.7 | 65.3 |
|  | SVM | 90.4 | 78.8 | 87.8 | 51.5 | 77.3 | 61.8 |
|  | NBC | 89.6 | 78.7 | 93.0 | 72.7 | 72.7 | 72.7 |
|  | AB | 89.2 | 84.4 | 91.9 | 64.3 | 81.8 | 72.0 |
|  | XGB | 88.6 | 78.8 | 94.8 | 88.2 | 68.2 | 76.9 |
|  | MLP | 86.3 | 80.5 | 90.1 | 58.6 | 77.3 | 66.7 |
|  | KNN | 88.4 | 67.4 | 84.9 | 44.7 | 77.3 | 56.7 |
|  | DT | 79.1 | 67.3 | 91.3 | 66.7 | 63.6 | 65.1 |

**AUC,** Area Under the Curve; **AP**, Average Precision; **F1**, F1 Score; **RF,** Random Forest; **LGBM,** Light Gradient Boosting Machine; **XGB,** eXtreme Gradient Boosting; **DT,** Decision Tree; **KNN,** K-Nearest Neighbors; **MLP,** Multi-Layer Perceptron; **AB,** AdaBoost; **SVM,** Support Vector Machine; **LR,** Logistic Regression; **NBC,** Naive Bayes Classifier.

**Table S2.** Sensitivity Analysis Using Routinely Available Clinical Variables and Laboratory Tests in the Internal Test Set and Short Time-Based External Validation Cohort.

|  | **Model** | **AUC**  **（%）** | **AP**  **（%）** | **Accuracy**  **（%）** | **Precision**  **（%）** | **Recall**  **（%）** | **F1**  **（%）** |
| --- | --- | --- | --- | --- | --- | --- | --- |
| **Training set** | RF | 97.3 | 96.4 | 93.3 | 98.3 | 95.3 | 94.3 |
|  | LGBM | 96.0 | 94.9 | 96.9 | 95.3 | 94.3 | 95.7 |
|  | XGB | 94.3 | 94.6 | 96.4 | 93.2 | 93.4 | 97.6 |
|  | DT | 93.4 | 94.5 | 93.2 | 95.3 | 94.5 | 93.2 |
|  | KNN | 97.4 | 97.3 | 89.7 | 55.6 | 98.6 | 71.1 |
|  | MLP | 96.6 | 94.7 | 95.7 | 77.7 | 93.8 | 85.0 |
|  | AB | 90.6 | 75.7 | 84.2 | 43.5 | 74.5 | 55.0 |
|  | SVM | 88.5 | 73.8 | 89.0 | 55.9 | 69.0 | 61.7 |
|  | LR | 84.8 | 72.0 | 90.5 | 63.6 | 61.4 | 62.5 |
|  | NBC | 83.6 | 70.0 | 89.5 | 58.8 | 62.1 | 60.4 |
| **Internal test set** | RF | 94.3 | 86.2 | 95.5 | 93.5 | 69.0 | 79.5 |
|  | LR | 91.9 | 81.3 | 91.6 | 65.9 | 69.0 | 67.4 |
|  | NBC | 96.4 | 87.6 | 93.1 | 68.6 | 83.3 | 75.3 |
|  | SVM | 90.2 | 81.8 | 91.3 | 61.4 | 83.3 | 70.7 |
|  | AB | 91.0 | 80.4 | 86.8 | 48.5 | 78.6 | 60.0 |
|  | XGB | 89.4 | 75.7 | 93.4 | 81.3 | 61.9 | 70.3 |
|  | LGBM | 90.7 | 77.2 | 93.1 | 80.6 | 59.5 | 68.5 |
|  | KNN | 83.9 | 69.3 | 86.2 | 47.0 | 73.8 | 57.4 |
|  | MLP | 80.3 | 73.0 | 91.3 | 65.1 | 66.7 | 65.9 |
|  | DT | 78.5 | 65.9 | 91.0 | 65.0 | 61.9 | 63.4 |
| **Short time‑based external validation cohort** | RF | 92.7 | 82.4 | 95.3 | 100.0 | 63.6 | 77.8 |
|  | LGBM | 88.8 | 75.7 | 94.8 | 93.3 | 63.6 | 75.7 |
|  | LR | 90.8 | 82.5 | 93.6 | 73.9 | 77.3 | 75.6 |
|  | SVM | 90.4 | 64.9 | 89.5 | 57.1 | 72.7 | 64.0 |
|  | NBC | 90.9 | 81.9 | 93.0 | 72.7 | 72.7 | 72.7 |
|  | AB | 91.5 | 80.9 | 90.7 | 60.7 | 77.3 | 68.0 |
|  | XGB | 90.0 | 76.9 | 94.8 | 93.3 | 63.6 | 75.7 |
|  | MLP | 78.5 | 70.8 | 91.3 | 66.7 | 63.6 | 65.1 |
|  | KNN | 79.4 | 67.9 | 83.7 | 41.7 | 68.1 | 51.7 |
|  | DT | 79.8 | 69.1 | 91.9 | 70.0 | 63.6 | 66.7 |

**AUC,** Area Under the Curve; **AP**, Average Precision; **F1**, F1 Score; **RF,** Random Forest; **LGBM,** Light Gradient Boosting Machine; **XGB,** eXtreme Gradient Boosting; **DT,** Decision Tree; **KNN,** K-Nearest Neighbors; **MLP,** Multi-Layer Perceptron; **AB,** AdaBoost; **SVM,** Support Vector Machine; **LR,** Logistic Regression; **NBC,** Naive Bayes Classifier.

**Table S3.** Hyperparameter Search Space for Machine Learning Models.

| **Model** | **Key Hyperparameters & Search Space** |
| --- | --- |
| **Random Forest** | n_estimators: [100,200,500,1000]; max_depth: [10,20, 30,None]; min_samples_split: [2,5, 10]; min_samples_leaf: [1, 2,4] |
| **LightGBM** | n_estimators: [100,200,500]; learning_rate: [0.01, 0.05, 0.1];max_depth: [5,10,15,-1]; num_leaves: [31, 63, 127] |
| **XGBoost** | n_estimators: [100,200,500]; learning_rate: [0.01, 0.05, 0.1];max_depth:[3,6,9,12];subsample: [0.8, 0.9, 1.0] |
| **Decision Tree** | max_depth: [5,10,15,20,None]; min_samples_split: [2, 5, 10];min samples leaf: [1,2.4] |
| **K-Nearest Neighbors** | n_neighbors:[3,5,7, 9,11]; weights: ['uniform' 'distance'] |
| **Multi-layer Perceptron** | hidden_layer_sizes:[(50,),(100,),(50,50),(100,50)]: activation:['rule', 'tanh']; alpha: [0.0001, 0.001, 0.01] |
| **AdaBoost** | n_estimators: [50,100,200]; learning_rate: [0.01, 0.1, 1.0] |
| **Support Vector Machine** | C: [0.1, 1, 10, 100]; kernel: ["inear, 'rbf']; gamma: ['scale, 'auto'] |
| **Logistic Regression** | C: [0.001,0.01,0.1, 1, 10,100]; penalty: [11, 2]; solver:['liblinear, 'saga'] |
| **Naive Bayes Classifier** | var_smoothing:[1e-9,1e-8, 1e-7, 1e-6, 1e-5] |

**Table S4.** Missing Data Summary for Predictor Variables.

| **Variable Group** | **Variable Name** | **Missing Count (n)** | **Missing Percentage (%)** |
| --- | --- | --- | --- |
| **Demographic characteristics** | Age | 0 | 0.0% |
|  | Male | 0 | 0.0% |
| **Medical history** | Preterm birth | 12 | 0.7% |
|  | Personal history of atopy | 20 | 1.2% |
|  | Personal history of wheezing | 12 | 0.7% |
|  | Breast feeding | 17 | 1.1% |
| **Clinical characteristics** | Fever | 0 | 0.0% |
|  | Cough | 0 | 0.0% |
|  | Wheezing | 0 | 0.0% |
|  | Dyspnea | 0 | 0.0% |
| **Laboratory indices** | White blood cell count | 34 | 2.1% |
|  | Absolute neutrophil count | 0 | 0.0% |
|  | Absolute lymphocyte count | 0 | 0.0% |
|  | Hemoglobin | 0 | 0.0% |
|  | Platelet | 0 | 0.0% |
|  | C-reactive protein | 0 | 0.0% |
|  | Procalcitonin | 0 | 0.0% |
|  | Serum ferritin | 0 | 0.0% |
|  | Iinterleukin-6 | 0 | 0.0% |
|  | Alanine aminotransferase | 0 | 0.0% |
|  | Aspartate aminotransferase | 0 | 0.0% |
|  | Creatine kinase | 0 | 0.0% |
|  | Creatine kinase myocardial band | 0 | 0.0% |
|  | Lactate dehydrogenase | 0 | 0.0% |
|  | Urea | 0 | 0.0% |
|  | Creatinine | 0 | 0.0% |
|  | Uric acid | 0 | 0.0% |
|  | Sodium | 0 | 0.0% |
|  | Calcium | 0 | 0.0% |
|  | Prothrombin time | 0 | 0.0% |
|  | Activated partial thromboplastin time | 0 | 0.0% |
|  | D-dimer | 0 | 0.0% |
|  | Antithrombin III | 0 | 0.0% |
|  | Immunoglobulin A | 0 | 0.0% |
|  | Immunoglobulin M | 0 | 0.0% |
|  | Immunoglobulin G | 0 | 0.0% |

**Table S5.** Distribution of RSV Diagnostic Methods Stratified by Ward Location.

| **RSV diagnostic method** | **Overall (n=1606)** | **GPW (n=1397)** | **PICU (n=209)** | **P** |
| --- | --- | --- | --- | --- |
| **Nucleic acid testing** | 1284 | 1110(79.5%) | 173(83.8%) | 0.262 |
| **Rapid antigen testing** | 322 | 287(20.5%) | 36(17.2%) |  |

RSV,Respiratory syncytial virus; GPW, general pediatric ward; PICU, pediatric intensive care unit.

**Table S6.** Univariable performance of dyspnea alone and performance of the Random Forest model after excluding predictors overlapping with PICU admission criteria.

| **Analysis** | **Dataset** | **AUROC** | **Recall (Sensitivity)** | **Specificity** | **Precision** | **F1** |
| --- | --- | --- | --- | --- | --- | --- |
| **Dyspnea alone (single binary predictor)** | Internal test set | 0.77 | 0.55 | 0.996 | 0.96 | 0.70 |
| **Reduced model (excluding dyspnea and wheezing)** | Internal test set | 0.87 | 0.68 | — | 0.77 | 0.71 |
| **Reduced model (excluding dyspnea and wheezing)** | Short time-based external validation cohort | 0.84 | 0.62 | — | 0.72 | 0.67 |

Note: The reduced model used the same hyperparameters as the primary model but excluded dyspnea and wheezing from the feature set. Specificity for the reduced model is not reported because the model outputs probabilities and requires a threshold to calculate binary specificity; the primary manuscript already reports calibration and decision curve analyses.

**Table S7.** Subgroup performance of the Random Forest model for predicting PICU admission.

| **Subgroup** | **N (overall)** | **N (test set)*** | **AUROC** | **Recall** | **F1** |
| --- | --- | --- | --- | --- | --- |
| **Age < 12 months** | 833 | 176 | 0.91 | 0.73 | 0.79 |
| **Age ≥ 12 months** | 773 | 146 | 0.93 | 0.78 | 0.82 |
| **Preterm birth (yes)** | 102 | 21 | 0.89 | 0.67 | 0.71 |
| **Preterm birth (no)** | 1504 | 301 | 0.94 | 0.79 | 0.83 |

PICU, pediatric intensive care unit; *Test set sample sizes are based on the 80/20 random split of the development cohort (n=1,606). The random seed was set to 42 for reproducibility.

**Fig S1.** Flow chart of enrollment.


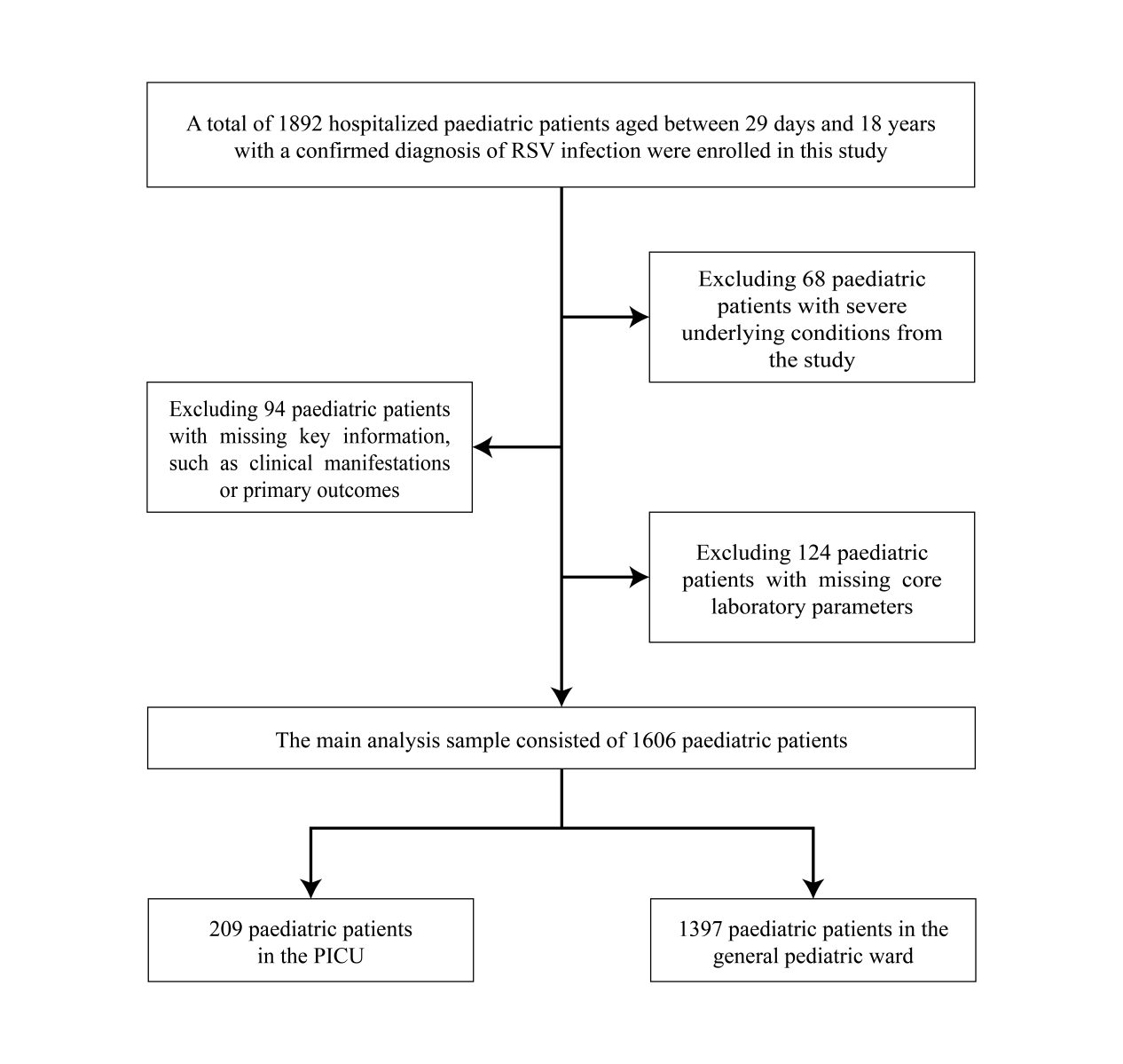


PICU, pediatric intensive care unit.

**Fig S2.** Performance Comparison of Ten Machine Learning Models During Training: Calibration Curves for Training, Internal Test, and Short Time-Based External Validation Sets.


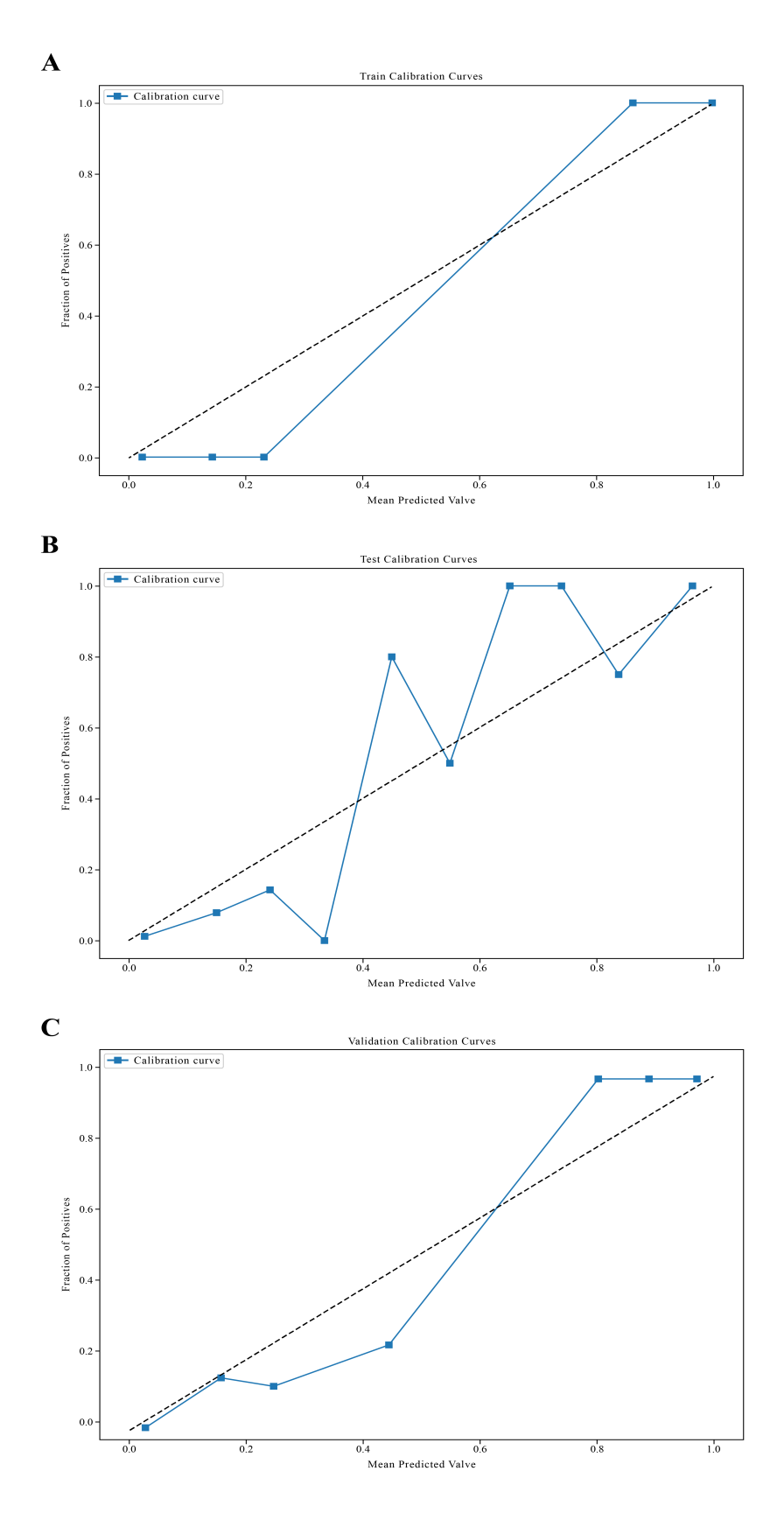

Supplement: Supplementary file 1 [file Supplementary_file_1.docx]
